# Semisynthesis, Characterization and Evaluation of New Adenosine Derivatives as Antiproliferative Agents

**DOI:** 10.3390/molecules23051111

**Published:** 2018-05-08

**Authors:** Francisco Valdés Zurita, Nelson Brown Vega, Margarita Gutiérrez Cabrera

**Affiliations:** 1 Institute of Chemistry of Natural Resources, University of Talca, 3460000 Talca, Chile; franciscovaldesz@hotmail.es; 2 Medical School, University of Talca, 3460000 Talca, Chile; nbrown@utalca.cl; 3 Programa de Investigación Asociativa en Cáncer Gástrico (PIA-CG), Universidad de Talca, 3460000 Talca, Chile

**Keywords:** nucleoside derivatives, adenosine, cancer, antiproliferative activity

## Abstract

We describe the semisynthesis and biological effects of adenosine derivatives, which were anticipated to function as agonists for the A_3_ receptor. Molecular docking was used to select candidate compounds. Fifteen nucleoside derivatives were obtained through nucleophilic substitutions of the *N*^6^-position of the nucleoside precursor 6-chloropurine riboside by amines of different origin. All compounds were purified by column chromatography and further characterized by spectroscopic and spectrometric techniques, showing moderate yield. These molecules were then evaluated for their antiproliferative activity in human gastric cancer cells expressing the A_3_ receptor. We found that the compounds obtained have antiproliferative activity and that new structural modifications can enhance their biological activity. The ADME (Absorption, Distribution, Metabolism and Excretion) properties of the most active compounds were also evaluated theoretically.

## 1. Introduction

Nucleosides are endogenous compounds that play critical roles in various cellular processes [1]. This multifunctionality renders these molecules interesting for both design and drug discovery [2]. This group of compounds encompasses analogues of the naturally occurring DNA and RNA bases, as well several derivatives of nucleosides and nucleotides of these bases.

**Figure 1:**
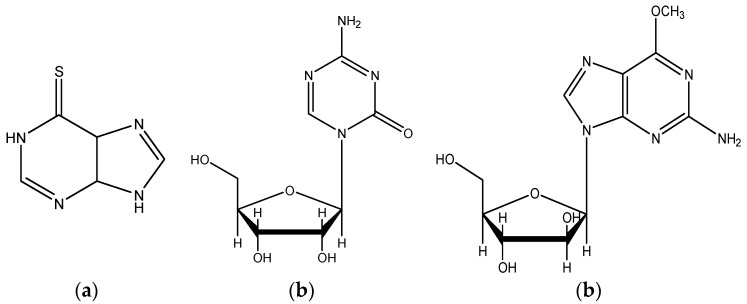
(**a**) 6-mercaptopurine, (**b**) azacitidine, and (**c**) nelarabine act as nucleic acid synthesis inhibitors and have been approved as anticancer drugs by the Food and Drug Administration (FDA).

Due to the ubiquitous nature and widespread functions of nucleoside derivatives (NDs), structural modifications can be interesting as tools for the generation of agonists or antagonists. Several reports have shown that a number of chemical modifications in nucleosides confer important improvements in their bioactivity. For example, NDs have shown antiviral and antibacterial activities [3,4,5], and some of them have also proven effective as anticancer agents [6,7]. Indeed, many nucleoside analogues have already been approved by the Food and Drug Administration (FDA) for cancer treatment. For example, the base analogue 6-mercaptopurine (Figure 1a) has been used to treat acute lymphoblastic leukemia, as well as various autoimmune disorders [8]. Likewise, azacytidine (Figure 1b), a pyrimidine analogue, has been used to treat myelodysplastic syndromes [9], and nelarabine (Figure 1c) was approved in 2005 for the treatment of T-cell lymphoblastic lymphoma/leukemia [10].

Among nucleosides, those containing purine bases are of great importance in medicinal chemistry. This medical potential has led to the organic synthesis and production of new purine derivatives in order to enhance their biological effects or improve yields when directly obtained from natural sources. Not surprisingly, derivatives and analogues of purine bases have been shown to possess a wide range of biological properties [5,11,12,13].

Adenosine, a natural nucleoside present in different organs and a known modulator of physiological and pathophysiological processes, can bind to at least four subtypes of G-protein-coupled receptors [14]. Chemical modifications of the nucleus of adenosine have produced compounds with interesting biological applications. For example, *N*^6^-focused structural modifications of adenosine-containing nucleosides have given rise to cytokinin nucleosides containing a hydrophilic ribofuranose moiety and a purine heterocyclic nucleus [5]. Previously, *N*^6^-substituted adenosines had been reported as natural products isolated from a cell culture of anise [15] in the endosperm liquid of fresh young coconut fruits [16] and as an abundant terpene nucleoside derived from *Mycobacterium tuberculosis* [17]. Also, *N*^6^-substituted-adenosines have been further modified in order to specifically target any one of the four receptors of adenosine (A_1_, A_2A_, A_2B_ and A_3_) [18,19], revealing antitumor and antiproliferative effects [20,21].

The assessment of adenosine and its derivatives as candidates for adjuvant anti-cancer therapy [22] has had a considerable impact on the treatment of certain types of tumors [23]. In this context, all subtypes of purinergic receptors (A_1_, A_2A_, A_2B_ and A_3_) could be exploited as potential targets for the development of new anti-tumor drugs [24]. In particular, the activation of signaling pathways dependent on A_3_ receptor activation can lead to reduced proliferation or induction of apoptosis in some tumor cells [24,25]. Nevertheless, the activation of the A_3_ receptor may also prove advantageous to cancer cells during their adaptation to hypoxia, a condition commonly observed in rapidly growing solid tumors [26].

Herein, we report the semisynthesis and chemical characterization of fifteen adenosine derivatives with different substituents at the *N*^6^-position. These substitutions were predicted to confer agonist activity on the A_3_ receptor. In addition, proliferative assays were used to assess the ability of these derivatives to impair proliferation of A_3_ receptor-expressing human gastric epithelial adenocarcinoma cells (AGS).

## 2. Results and Discussion

### 2.1. Molecular Docking of Adenosine Derivatives

The Gaussview 5.0 (Semichem Inc., Shawnee, KS, USA) and the Maestro suite programs (Schrödinger, LLC, New York, NY, USA) were used for the in silico design of adenosine analogs from the precursor 6-chloropurine-riboside. This procedure gave rise to a set of 284 molecules that included compounds with modifications at the ribose ring, as well as compounds in which the *N*^6^ position of the purine ring was substituted by aliphatic and aromatic amines. Conformations of the most stable structures were obtained, which represent those with less repulsion. Following docking, a total of 1572 molecular coupling conformers were obtained. Subsequently, a filter of selection was applied based on the interactions and/or contacts between the compounds and the amino acid residues of interest present in the active site of the A_3_ receptor. After application of the pose-filter script, only 42 conformers were obtained, corresponding to fourteen compounds. These compounds were then compared to the commercially available agonist (IB-MECA) and antagonist (MRS-1523).

The most favorable energy values for each ligand-A_3_ receptor binding were identified, selected, and registered (Table 1). As an auxiliary discrimination method, free energies of interaction were calculated through MMGB-SA (Molecular Mechanics–Generalized Born Surface Area) for each compound, including an agonist and an antagonist as references.

**Figure 2:**
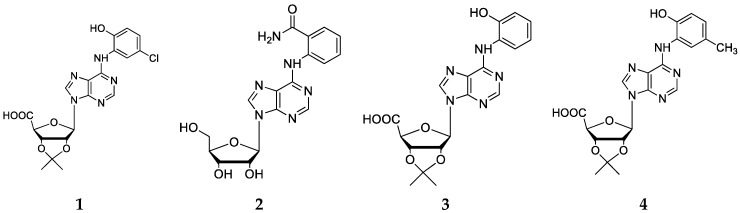

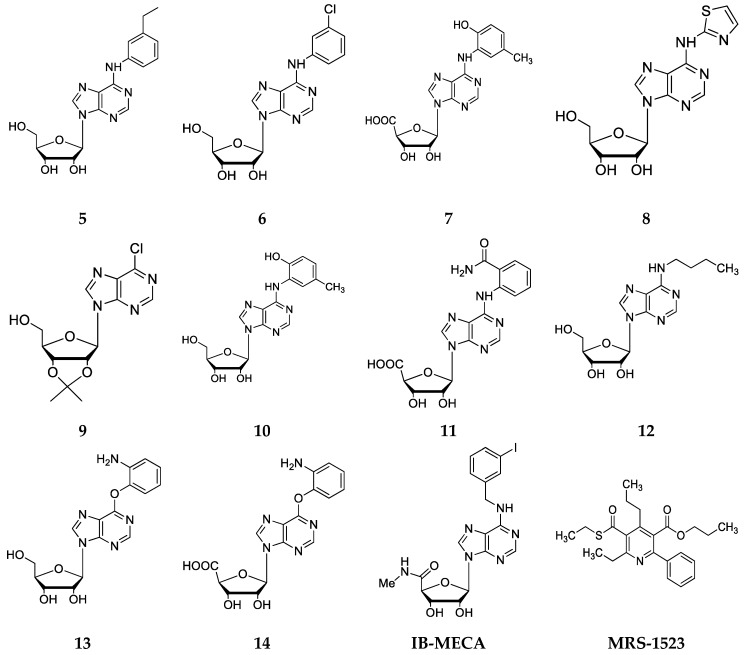
Chemical structures of selected compounds derived from in silico analyses.

Post-docking selected compounds were grouped and numbered in Figure 2. These compounds showed structural diversity, with several modifications in the ribose region of the adenosine nucleus (protection of the 2′-OH and 3′-OH groups; oxidation of the 5′-OH group to a carboxylic acid) and different aromatic substitutions at the *N*^6^-position.

**Figure 3:**
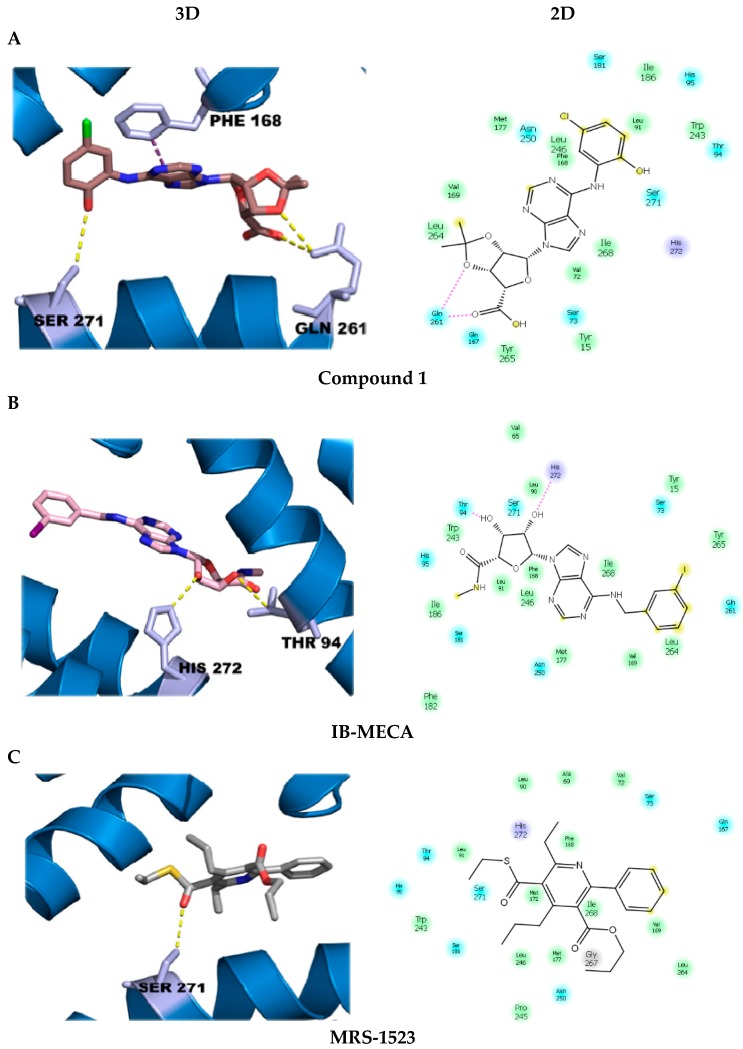
3D and 2D interactions of the semi-synthesized compound **1** (**A**), the selective commercial selective agonist, IB-MECA (*N*^6^-(3-Iodobenzyl) adenosine-5′-N-methyluronamide) (**B**), and the commercial selective antagonist, MRS-1523 (**C**), with residues at the active site of the A_3_ receptor.

Molecular dockings were visualized through the PyMOL software, identifying interactions of some ligands with the active site of the A_3_ receptor (Figure 3). Compound **1** displayed the highest binding energy compared to the other compounds, including the selective agonist IB-MECA (*N*^6^-(3-Iodobenzyl) adenosine-5′-*N*-methyluronamide). Figure 3A shows some of the interactions established by compound 1 with important amino acid residues of the active site of the A_3_ receptor. As a comparison, Figure 3B, C show the interactions displayed by the selective agonist and the selective antagonist, B-MECA and MR-1523, respectively.

Similar results to those reported by Valdés et al. [27] were obtained. The fourteen compounds presented interactions pi–pi stacking between the purine ring and Phe 168, and a ring-type interaction with Asn 250. *N*^6^-substituted compounds also displayed three hydrogens bonds between Ser 271 and the oxygens of 2′-OH and 3′-OH groups. However, some compounds presented hydrogen bonds between the Gln 261 and the 5′-COOH group. For example, the docking for compound **1** showed hydrogen bonds, hydrophobic and mild polar interactions, as well as pi–pi stacking interactions. The residue of Ser 271 displayed a hydrogen bond interaction with the oxygen of the OH-Ar substitution group and two hydrogen bonds with Gln 261. The Phe 168 showed pi–pi interaction with the purine ring.

The interactions found for the selective agonist IB-MECA confirmed previous reports [27,28,29]. It is important to mention that most of the compounds that were able to form hydrogen bonds between the 3′-*O*-isopropylidene group and Ser 271 acted as agonist molecules [29]. In addition, when the compounds showed pi–pi stacking interactions with Phe 168 residue, these interactions were indicative of increased agonist-receptor binding affinity [30].

Likewise, the interactions shown by the selective antagonist for the A_3_ receptor, known as MRS-1523, depended on a single hydrogen bond between the thiocarbonyl group and the Ser 271 residue, allowing the compound to be recognized as an antagonist.

Therefore, the interactions displayed by compound **1** were energetically more favorable than those shown by the selective agonist IB-MECA. When compared to the antagonist MRS-1523, the interactions were weaker in proportion and less energetically favorable than compound **1**.

Regarding the binding energies of the different compounds to the active site of the A_3_ receptor, these were difficult to assert, which led us to perform free energy calculations of interactions between two systems (ligand-protein). This methodology is called MMGB-SA (molecular mechanics-generalized surface area) and was used as an auxiliary discrimination method [31,32]. However, there were not significant differences between the energy values presented by the compounds and the energies of the agonist (IB-MECA), which would be a positive indicator in the search for new agonists with the same or better coupling shape and affinity for the A_3_ receptor.

### 2.2. Chemical Synthesis

**Figure 4:**
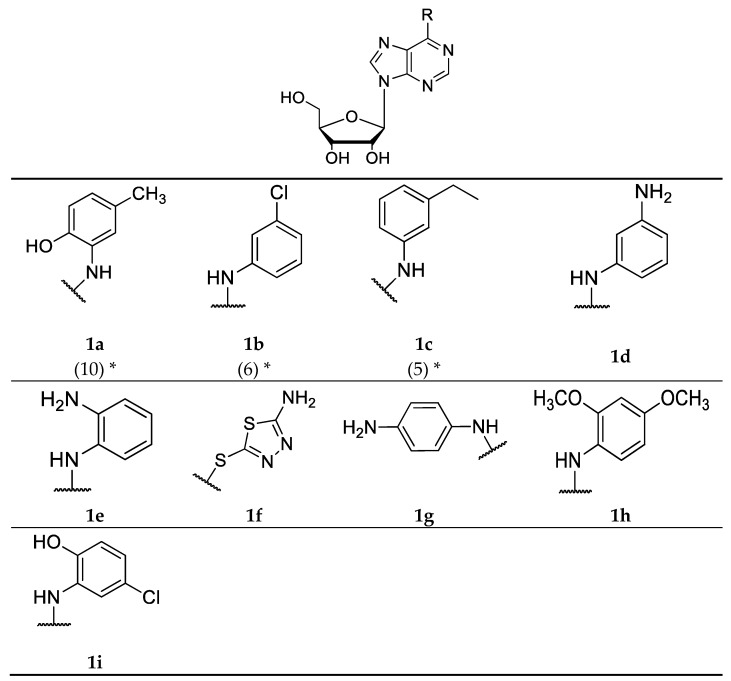
Structures of adenosine derivatives that belong to the series 1. (* Compounds suggested by molecular docking).

**Figure 5:**
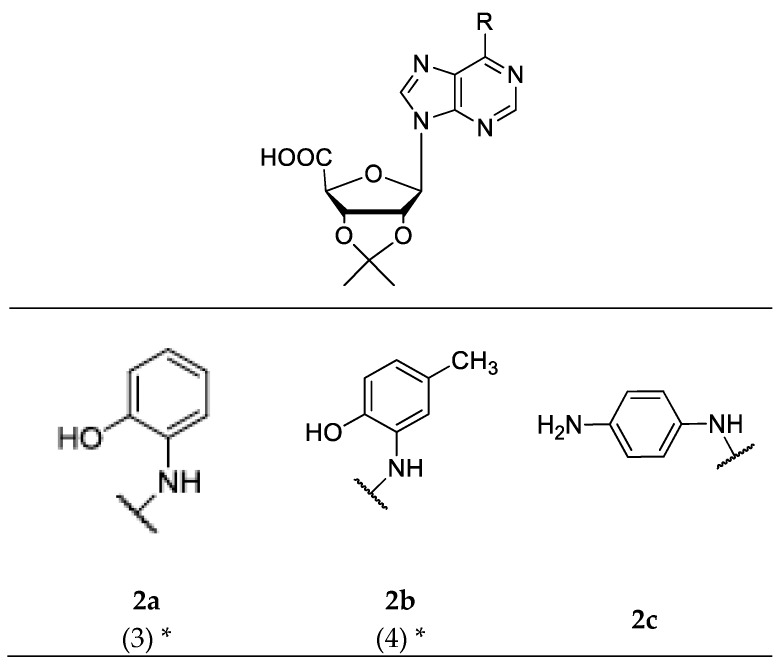

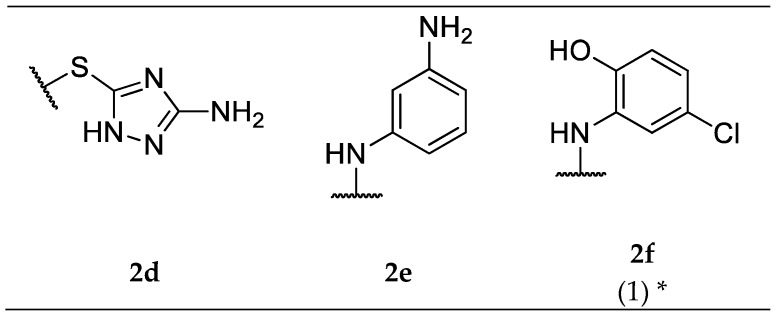
Structures of adenosine derivatives that belong to series 2. (* Compounds suggested by molecular docking).

Next, we synthesized a subset of adenosine derivatives with different patterns of substitution at the *N*^6^ position of the aromatic ring. Each substitution was carried out using amines of different origin according to the bioinformatic procedures previously described [27]. In total, six of the compounds proposed by molecular docking were synthesized. In addition, we also synthesized compounds that were not selected by molecular docking for obtaining greater structural diversity of adenosine derivative compounds. Series **1** (Figure 4a–i) was obtained by nucleophilic substitutions with aromatic and aliphatic amines, with yields between 43% and 79%. The series **2** (Figure 5a–f) was obtained by a modification of the precursor at the diol (2′-OH and 3′-OH groups) of the ribose and by a total oxidation of 5′-OH group, followed by a nucleophilic substitution with amines at the *N*^6^ position of the precursor, with yields between 43% and 98%. All compounds were isolated by column chromatography. The structures of all derivatives were confirmed by IR and NMR spectroscopy, showing signals that were characteristic of aromatic protons near to heteroatom and aliphatic atom, being identified in the ^1^H-NMR. The IR spectra showed signals of 3500 cm^−1^ that were characteristic of hydroxyl groups and signals between 1640 and 1560 cm^−1^ for primary amines, and 1500 cm^−1^ for secondary amines.

In the particular case of compounds **1f** and **2d**, the substitution was performed between the chlorine atom of the nucleoside and the thiol group of the amine, this because of the higher reactivity and nucleophilia of the thiol group compared to the amine group.

The structural diversity of both synthesized series is presented in Figure 4 and Figure 5, where each “R” represents a substituent that was coupled to the *N*^6^-position of the purine ring. It is important to mention that series 1 and 2 preserve a common structural core, formed by a ribose (with or without modifications) and a purine ring.

### 2.3. Biological Activity of Adenosine Derivatives

**Figure 6:**
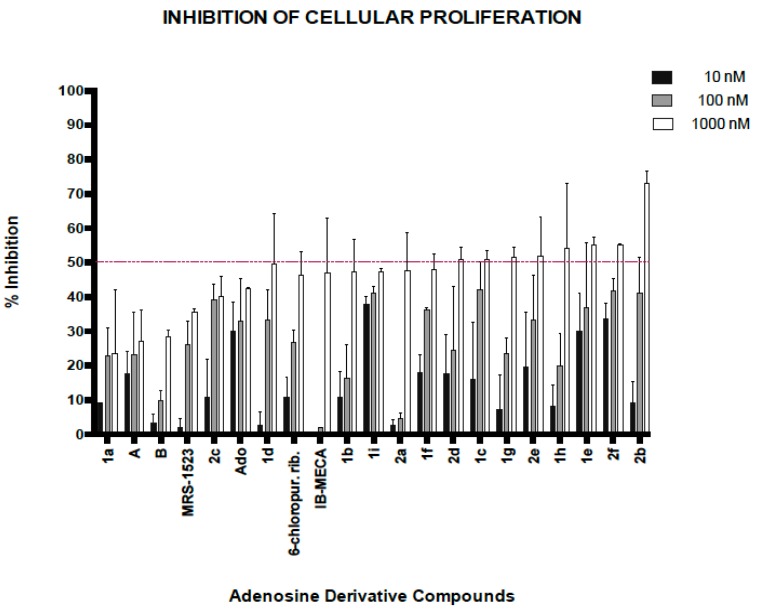
The ability of adenosine derivatives to inhibit the proliferation AGS cells (gastric adenocarcinoma) was assessed. Three concentrations (10 nM, 100 nM and 1000 nM) for each compound were tested. Compounds of the series 1 (**1a**–**1h**), 2 (**2a**–**2f**), the selective agonist (IB-MECA) and antagonist (MRS-1523), the protected adenosine (A) modified 5′-OH group and protected on the ribose ring (B) (see Figure 7), adenosine (Ado) and 6-chloropurine riboside were all tested. The values represent the percentage of inhibition with respect to the negative control used (0.2% DMSO) ± standard deviation (SD). Interestingly, most of the compounds tested display better percentages of inhibition at concentrations of 10 and 100 nm when compared to the selective agonist (IB-MECA).

All derivatives were tested for their ability to impair proliferation of A_3_-expressing AGS gastric cancer cells. For each compound, final concentrations of 10, 100 and 1000 nM were tested for 72 h. The percentage of inhibition of cell proliferation was determined and compared to the inhibitory effects of the positive control IB-MECA. The results are shown in Figure 6.

Based on their antiproliferative activity, the most interesting compounds were **1e**, **1h**, **2b** and **2f**. These compounds displayed the highest degrees of inhibition with the lowest concentrations. It is important to mention that the compound IB-MECA showed antiproliferative activity only at the highest concentration (1000 nM) tested, which is in stark contrast with its efficacy shown in other cell lines [33]. Overall, most of the structures obtained by semisynthesis showed improvements in biological activity (i.e., antiproliferative activity), corroborating the hypothesis that modifications at the *N*^6^-position influence the biological effects of adenosine derivatives.

Taking the in silico and biological data together, two compounds showed a good correlation between computational calculations (in silico) and results obtained by cell proliferation assays (in vitro). Thus, docking analyses first revealed that compounds **2b** and **2f** had better binding energies to the active site of the A_3_ receptor (∆G_b_ > −75 Kcal/mol). After testing these compounds in AGS cells, they also showed better inhibitory effects at concentrations of 100 nM and 1000 nM [20,24].

Some selected molecular properties were predicted using Molinspiration [34]. Lipinski’s rule of five is commonly used to predict oral bioavailability of potential lead or drug molecules. According to this, a candidate molecule will likely be active orally, if: (i) the molecular weight is under 500; (ii) the calculated octanol/water partition coefficient (Log P) is <5 L; (iii) it has fewer than five hydrogen bond donors (OH and NH groups) and; (iv) it has less than ten hydrogen bond acceptors (notably N and O) [34]. The molecular properties of the most active adenosine derivatives (**1e**, **1h**, **2b** and **2f**) were calculated using the Molinspiration cheminformatics software [34]. The parameters obtained are presented in Table 2. Only one example of violation of the Lipinski’s rules [35] was observed in the four compounds analyzed (**1e**, **1h**, **2b**, **2f**), namely the number of hydrogen bond acceptors. The Log P values (partition coefficients, an indicator of lipophilicity) of all compounds were found to be less than 5, suggesting good permeability across cell membranes. Similarly, the molecular weight of the selected derivatives was found to be less than 500. Thus, these molecules were predicted to be easily transported, diffused and absorbed across biological membranes. On the other hand, the number of hydrogen bond donors (NH and OH) in compound **1h**, **2b**, and **2f** were also in accordance with Lipinski’s rules (i.e., less than 5). Overall, the compound **2f** is most likely to be orally active as it obeys most of Lipinski’s rules.

The topological polar surface area (TPSA) is a useful parameter for predicting drug transport properties. It is defined as the sum of surfaces of polar atoms (normally oxygens, nitrogens and attached hydrogens) in a molecule. TPSA can be correlated with hydrogen bonding, being a good indicator of bioavailability of drugs. Thus, this parameter has been shown to correlate very well with human intestinal absorption, in vitro monolayer permeability, and blood–brain barrier penetration. All TPSA values of the adenosine derivatives were observed in order of 150 Å, that is, below the limit of 160 Å. From the results, we can conclude that the compounds **1e**, **1h**, **2b**, **2f** can be used as starting points in the development of potential drugs.

## 3. Materials and Methods

### 3.1. Molecular Docking’s Study of Adenosine Analogues

All the ligand structures were constructed using Gaussview 5.0 (Semichem Inc., Shawnee, KS, USA). The glide tool from Maestro suite (Glide, version 5.7, Schrödinger, LLC, New York, NY, USA) [36] was also used. These software were used to perform the molecular docking of the compounds designed, applying a hierarchical series of filters that allow to find possible locations of the ligand in the region of the active site of the human receptor A_3_. Information of conformational, positional, and orientational space of the ligands coupled to the active site was obtained under a force field OPLS-SAA (All-Atom Optimized Potentials for Liquid Simulations) [37].

### 3.2. Selection of the Best Conformers

After performing molecular docking, the pose-filter script was used to select the pool of conformers that had some interaction with amino acid residues considered important by different authors [28,37]. To select the best conformers for each ligand, criteria used by Valdes et al. were applied [27].

### 3.3. Chemistry

Melting points were measured using a Büchi apparatus and are uncorrected. The purity of compounds was checked through analytical TLC (thin layer chromatography) on silica gel plates (Merck 60 F254, KGaA, Darmstadt, Germany). Compounds were purified by column chromatography when necessary. Chemicals were bought from Aldrich (KGaA, Darmstadt, Germany) and used without further purification. ^1^H and ^13^C-NMR spectra (400.1 MHz for proton and 100.6 MHz for carbon) were recorded in an AM-400 spectrometer (Bruker, Rheinstetten, Germany), using DMSO-*d*_6_ as solvent. Tetramethylsilane (TMS) was used as an internal standard. Chemical shifts (*δ*) and J values are reported in ppm and Hz, respectively; relative to the solvent peak DMSO-*d*_6_ 2.5 ppm for protons and 39.7 ppm for carbon atoms. Signals are designated as follows: s, singlet; d, doublet; dd, doublet of doublets; t, triplet; m, multiplet; br.s, broad singlet. IR spectra (KBr pellets, 500–4000 cm^−1^) were recorded on a NEXUS 670 FT-IR spectrophotometer (Thermo Nicolet, Madison, WI, USA).

### 3.4. Synthesis

All adenosine derivatives were prepared by condensing 6-chloropurine riboside (6-chloro-9-*b*-d-ribofuranosyl-9*H*-purine) with amines of different nature, including amines and diamines with straight and branched chains, as well as aromatic amines by nucleophilic aromatic substitution. This gave rise to two series of compounds:

Series 1: these derivatives were obtained by nucleophilic aromatic substitution (S_N_Ar) in absolute ethanol, using *N*,*N*-diisopropylethylamine (DIPEA) as Lewis acid (Figure 8).

The derived nucleosides were obtained through semisynthetic methods described previously by Ottria et al. [12]. To a solution of 6-chloropurine riboside (0.35 mmol) in absolute EtOH or DMF, DIPEA (1.05 mmol) and the appropriate amine (4.5 mmol) were added. The mixture was stirred at 80 °C for 8 h and then cooled down to room temperature. The solvent was removed by filtration or under vacuum to leave a residue that was analyzed by TLC. The residue was washed with hexane, dried and purified by SiO_2_ column chromatography (CH_2_Cl_2_-MeOH, 97:3). In some cases, the addition of dry Et_2_O was used to precipitate DIPEACl (*N*,*N*-Diisopropylethylamine chloride), which was then filtered off. The crude residue obtained after evaporation was purified by column chromatography.

*2-Hydroxymethyl-5-[6-(2-hydroxy-5-methyl-phenylamino)-purin-9-yl]-tetrahydro-furan-3,4-diol* (**1a**): was prepared following the above describe procedure starting from 6-chloropurine riboside and 2-amine-4-methylphenol. Yellow solid 50.9% yield; m.p. 237–238 °C. ^1^H-NMR (DMSO-*d*_6_, 400.1 MHz) *δ*: 9.96 (s, 1H, NH); 8.58 (s, 1H, OH); 8.53 (s, 1H, CH-Ar purine); 8.44 (s, 1H, CH-Ar purine); 8.13 (d, *J* = 4.00 Hz, 1H, CH-Ar); 6.82 (m, 1H, CH-Ar); 6.74 (m, 1H, CH-Ar); 5.96 (d, *J* = 6.00 Hz, 1H, CH-1′); 5.48 (d, *J* = 6.11 Hz, 1 H, 2′-OH); 5.22 (m, 2H, CH_2_-5′); 4.64 (q, *J* = 5.83 Hz, 1H, CH-2′); 4.19 (m, 1H, CH-3′);3.99 (q, *J* = 3.55 Hz, 1H, CH-4′); 3.64 (m, 2H, CH_2_-5′); 2.25 (s, 3H, CH_3_-Ar). ^13^C–NMR (DMSO-*d*_6_, 100.6 MHz) *δ*: 152.6, 152.3, 149.3, 145.6, 141.6, 128.3, 127.2, 124.6, 122.3, 120.9, 115.5, 88.4, 86.3, 74.1, 70.9, 21.1. IR (KBr) λ/cm^−1^ 3358, 3117, 2926, 2857, 1636, 1476, 1216.Anal. Cal. C_17_H_19_N_5_O_5_: C = 54.64%, H = 5.09%, N = 18.75%.

*2-[6-(3-Chloro-phenylamino)-purin-9-yl]-5-hydroxymethyl-tetrahydro-furan-3,4-diol* (**1b**): was prepared following the above describe procedure starting from 6-chloropurine riboside and chloroaniline. Yellow solid 50.1% yield; m.p. 190–193 °C. ^1^H–NMR (DMSO-*d*_6_, 400.1 MHz) *δ*: 10.14 (s, 1H, NH); 8.59 (s, 1H, CH-Ar purine); 8.47 (s, 1 H, CH-Ar purine); 8.20 (s, 1H, CH-Ar); 7.90 (d, *J* = 8.07 Hz, 1H, CH-Ar); 7.33 (t, *J* = 7.83 Hz, 1H, CH-Ar); 7.08 (d, *J* = 7.83 Hz, 1H, CH-Ar); 5.98 (d, *J* = 5.98 Hz, 1H, CH-1′); 5.48 (d, *J* = 5.38 Hz, 1H, 2′-OH); 5.23 (d, *J* = 6.85 Hz, 2H, 3′-OH, 5′-OH); 4.65 (d, *J* = 4.89 Hz, 1H, CH-2′); 4.18 (s, 1H, CH-3′); 3.99 (s, 1H, CH-4′); 3.64 (m, 2H, CH_2_-5′). ^13^C–NMR (DMSO-*d*_6_, 100.6 MHz) *δ*: 151.7, 151.8, 149.7, 141.2, 141.0, 132.8, 130.0, 122.1, 120.5, 119.9, 118.9, 87.8, 85.8, 73.6, 70.5, 61.5. IR (KBr) λ/cm^−1^ 3441, 2923, 2860, 1641, 1482, 1219.Anal. Cal. C_16_H_16_ClN_5_O_4_: C = 50.82%, H = 4.25%, Cl = 9.38%, N = 18.53%. The spectroscopic dates were concordance with literature reported [38,39].

*2-[6-(3-Ethyl-phenylamino)-purin-9-yl]-5-hydroxymethyl-tetrahydro-furan-3,4-diol* (**1c**): was prepared following the above describe procedure starting from 6-chloropurine riboside and 3-ethyl aniline. White solid 54.4% yield; m.p. 181–184 °C. ^1^H–NMR (DMSO-*d*_6_, 400.1 MHz) *δ*: 9.81 (s, 1H, NH); 8.53 (s, 1H, CH-Ar purine); 8.40 (s, 1H, CH-Ar purine); 7.82 (s, 1H, CH-Ar); 7.20 (t, *J* = 8.07 Hz, 1H, CH-Ar); 6.89 (d, *J* = 7.46 Hz, 1H, CH-Ar); 7.08 (d, *J* = 7.83 Hz, 1H, CH-Ar); 5.97 (d, *J* = 5.87 Hz, 1H, CH-1′); 5.48 (s, 1H, 2′-OH); 5.25 (m, 2H, 3′-OH, 5′-OH); 4.61 (s, 1H, CH-2′); 4.18 (s, 1H, CH-3′); 3.99 (d, *J* = 2.93 Hz, 1H, CH-4′); 3.65 (m, 2H, CH_2_-5′);2.60 (q, *J=* 7.54 Hz, 2H, CH_2_-1′′); 1.21 (t, *J=* 7.52 Hz, 3H, CH_3_-2″).^13^C–NMR (DMSO-*d*_6_, 100.6 MHz) *δ*:152.7, 152.4, 149.8, 144.4, 141.1, 139.9, 128.7, 122.8, 120.8, 120.8, 118.9, 88.3, 86.3, 74.1, 71.0, 62.0, 28.8, 15.9. IR (KBr) λ/cm^−1^ 3331, 2970, 2921, 1647, 1592, 1545, 1378, 1218. Anal. Cal. C_18_H_21_N_5_O_4_: C = 58.16%, H = 5.65%, N = 18.85%. The spectroscopic dates were concordance with literature reported [40].

*2-[6-(3-Amino-phenylamino)-purin-9-yl]-5-hydroxymethyl-tetrahydro-furan-3,4-diol* (**1d**): was prepared following the above describe procedure starting from 6-chloropurine riboside and 1,3-phenylendiamine. Solid amorphous gray, 71.8% yield; m.p. 198–200 °C. ^1^H–NMR (DMSO-*d*_6_, 400.1 MHz) *δ*: 9.54 (s, 1H, NH); 8.49 (s, 1H, CH-Ar purine); 8.35 (s, 1H, CH-Ar purine); 7.21 (d, *J* = 1.83 Hz, 1H, CH-Ar); 6.96 (m, 2H, CH-Ar); 6.29 (dt, *J* = 2.00 Hz, *J*_3_ = 7.00 Hz, 1H, CH-Ar); 5.94 (d, *J* = 5.99 Hz, 1H, CH-1′); 5.47 (d, *J* = 6.11 Hz, 1H, 2′-OH); 5.30 (m, 1H, 3′-OH); 5.20 (d, *J* = 4.65 Hz, 1H, 5′-OH); 5.00 (s, 2H, NH_2_); 4.63 (q, *J* = 5.91 Hz, 1H, CH-2′); 4.17 (m, 1H, CH-3′); 3.98 (q, *J* = 3.34 Hz, 1H, CH-4′); 3.63 (m, 2H, CH_2_-5′). ^13^C–NMR (DMSO-*d*_6_, 100.6 MHz) *δ*: 153.55, 152.80, 149.60, 149.22, 140.92, 140.38, 129.12, 120.75, 109.79, 109.72, 107.30, 88.33, 86.32, 74.04, 71.02, 62.04. IR (KBr) λ/cm^−1^ 3415, 3332, 3147, 2924, 2861, 1642, 1546, 1477, 1215. Anal. Cal. C_16_H_18_N_6_O_4_: C = 53.57%, H = 5.02%, N = 23.44%.

*2-[6-(2-Amino-phenylamino)-purin-9-yl]-5-hydroxymethyl-tetrahydro-furan-3,4-diol* (**1e**): was prepared following the above describe procedure starting from 6-chloropurine riboside and 1,2-diphenylendiamine. Yellow solid 62.9% yield; m.p. 201–205 °C. ^1^H–NMR (DMSO-*d*_6_, 400.1 MHz) *δ*: 9.10 (s, 1H, NH); 8.46 (s, 1H, CH-Ar purine); 8.20 (s, 1H, CH-Ar purine); 7.23 (dd, *J* = 7.83 Hz, *J* = 0.98 Hz, 1H, CH-Ar); 6.96 (m, 1H, CH-Ar); 6.77 (dd, *J* = 7.95 Hz, *J* = 1.10 Hz, 1H, CH-Ar); 6.59 (m, 1H, CH-Ar); 5.92 (d, *J* = 6.11 Hz, 1H, CH-1′); 5.46 (d, *J* = 5.87 Hz, 1H, 2′-OH); 5.36 (m, 1H, 3′-OH), 5.20 (d, *J* = 4.16 Hz, 1H, 5′-OH); 4.63 (q, *J* = 5.54 Hz, 1H, CH-2′); 4.17 (d, *J* = 3.18 Hz, 1H, CH-3′); 3.98 (q, *J* = 3.42 Hz, 1H, CH-4′); 3.69 (m, 1H, CH_2_-5′); 3.56 (m, 1H, CH_2_-5′); 3.35 (s, 2H, NH_2_). ^13^C–NMR (DMSO-*d*_6_, 100.6 MHz) *δ*: 161.3, 159.5, 150.6, 150.2, 134.7, 130.8, 126.9, 124.5, 120.9, 116, 2, 110.6, 88.3, 86.1, 73.3, 70.9, 61.6. IR (KBr) λ/cm^−1^ 3375, 3335, 3272, 2944, 2918, 1640, 1494, 1215. Anal. Cal. C_16_H_18_N_6_O_4_: C = 53.58%, H = 5.02%, N = 23.44%.

*2-[6-(5-Amino-[1,2,4]thiadiazol-2-ylsulfanyl]-5-hydroxymethyl-tetrahydro-furan-3,4-diol* (**1f**): was prepared following the above describe procedure starting from 6-chloropurine riboside and 2-amine-5-mercaptothiadiazole. Solid amorphous white, 70.5% yield; m.p. 220–223 °C. ^1^H–NMR (DMSO-*d*_6_, 400.1 MHz) *δ*: 8.83 (s, 1H, CH-Ar purine); 8.75 (s, 1H, CH-Ar purine); 7.65 (s, 2H, NH_2_); 5.99 (d, *J* = 5.38 Hz, 1H, CH-1′); 5.54 (d, *J* = 4.40 Hz, 1H, 2′-OH); 5.24 (s, 1H, 3′-OH); 5.07 (s, 1H, 5′-OH); 4.59 (m, 1H, CH-2′); 4.20 (m, 1H, CH-3′), 3.97 (d, *J* = 3.42 Hz, 1H, CH-4′); 3.70(m, 2H, CH_2_-5′). ^13^C–NMR (DMSO-*d*_6_, 100.6 MHz) *δ*: 173.6, 156.4, 152.2, 149.7, 144.9, 141.0, 131.1, 88.4, 86.2, 74.4, 70.6, 61.6. IR (KBr) λ/cm^−1^ 3511, 3389, 3112, 2955, 2929, 1644, 1566, 1219. Anal. Cal. C_12_H_13_N_7_O_4_S_2_: C = 37.56%, H = 3.39%, N = 25.56%, S = 16.69%.

*2-[6-(4-Amino-phenylamino)-purin-9-yl]-5-hydroxymethyl-tetrahydro-furan-3,4-diol* (**1g**): was prepared following the above describe procedure starting from 6-chloropurine riboside and 1,4-diphenylendiamine. Solid gray, 52.0% yield; m.p. 250–253 °C. ^1^H–NMR (DMSO-*d*_6_, 400.1 MHz) *δ*: 9.46 (s, 1H, NH); 8.42 (s, 1H, CH-Ar purine); 8.25 (s, 1H, CH-Ar purine); 7.42 (d, *J* = 8.31 Hz, 2H, CH-Ar); 6.50 (d, 2H, CH-Ar); 5.93 (d, *J* = 5.87 Hz, 1H, CH-1′); 5.48 (s, 1H, 2′-OH); 5.34 (s, 1H, 3′-OH), 5.19 (s, 1H, 5′-OH); 4.87 (s, 2H, NH_2_); 4.62 (s, 1H, CH-2′); 4.16 (s, 1H, CH-3′); 3.98 (s, 1H, CH-4′); 3.69 (m, 1H, CH_2_-5′); 3.53 (m, 1H, CH_2_-5′). ^13^C–NMR (DMSO-*d*_6_, 100.6 MHz) *δ*: 152.9, 152.5, 149.2, 145.3, 140.5, 128.6, 123.7, 114.1, 120.4, 88.4, 86.3, 71.1, 73.9, 62.1. IR (KBr) λ/cm^−1^ 3404, 3338, 3224, 2918, 2867, 1651, 1512, 1477, 1218. Anal. Cal. C_16_H_18_N_6_O_4_: C = 53.58%, H = 5.02%, N = 23.44%.The spectroscopic dates were concordance with literature reported [41].

*2-[6-(2,4-Dimethoxy-phenylamino)-purin-9-yl]-5-hydroxymethyl-tetrahydro-furan-3,4-diol* (**1h**): was prepared following the above describe procedure starting from 6-chloropurine riboside and 2,4-dimethoxyaniline. Solid gray, 63.1% yield; m.p. 173–176 °C. ^1^H–NMR (DMSO-*d*_6_, 400.1 MHz) *δ*: 8.57 (d, J = 1.22 Hz, 1H, NH); 8.42 (d, *J* = 2.20 Hz, 1H, CH-Ar purine); 8.27 (d, *J* = 2.20 Hz, 1H, CH-Ar purine); 7.84 (dd, *J* = 8.68 Hz, *J* = 1.59 Hz, 1H, CH-Ar); 6.67 (d, *J* = 2.20 Hz, 1H, CH-Ar); 6.53 (m, 1H, CH-Ar); 5.93 (dd, *J* = 5.99 Hz, *J* = 2.08 Hz, 1H, CH-1′); 5.47 (d, *J* = 4.40 Hz, 1H, 2′-OH); 5.33 (s, 1H, 3′-OH); 5.19 (s, 1H, 5′-OH); 4.64 (d, *J* = 3.67 Hz, 1H, CH-2′); 4.16 (s, 1H, CH-3′); 3.97 (s, 1H, CH-4′); 3.82 (d, *J* = 2.20 Hz, 3H, -OCH_3_); 3.76 (d, *J* = 2.45 Hz, 3H, -OCH_3_); 3.61 (m, 2H, CH_2_-5′). ^13^C–NMR (DMSO-*d*_6_, 100.6 MHz) *δ*: 157.55, 153.26, 152.92, 152.58, 121.05, 149.37, 141.12, 124.95, 120.60, 104.74, 99.43, 88.46, 86.35, 74.02, 71.04, 62.05, 56.28, 55.82. IR (KBr) λ/cm^−1^ 3392, 2941, 2832, 1626, 1540, 1426, 1286, 1212, 1046. Anal. Cal. C_18_H_21_N_5_O_6_: C = 53.55%, H = 5.21%, N = 17.35%.

*2-[6-(5-Chloro-2-hydroxy-phenylamino)-purin-9-yl]-5-hydroxymethyl-tetrahydro-furan-3,4-diol* (**1i**): was prepared following the above procedure, starting from 6-chloropurine riboside and 2-amine-4-chlorophenol. Brown solid, 79.2% yield; m.p. 214–218 °C. ^1^H–NMR (DMSO-*d*_6_, 400.1 MHz) *δ*: 10.82 (s, 1H, OH-Ar); 8.60 (s, 1H, CH-Ar purine); 8.50 (s, 1H, CH-Ar purine); 8.48 (s, 1H, CH-Ar); 7.04 (m, 1H, CH-Ar); 6.94 (d, *J* = 8.56 Hz, CH-Ar); 5.98 (d, *J* = 5.87 Hz, 1H, CH-1′); 5.55 (d, *J* = 5.62 Hz, 1H, 2′-OH); 5.26 (d, *J* = 4.40 Hz, 2H, 3′-OH, 5′-OH); 4.63 (q, *J* = 5.14 Hz, 1H, CH-2′); 4.15 (m, 1H, CH-3′); 3.96 (d, *J* = 3.18 Hz, 1H, CH-4′); 3.64 (m, 2H, CH_2_-5′). ^13^C–NMR (DMSO-*d*_6_, 100.6 MHz) *δ*: 152.5, 151.9, 149.6, 146.5, 141.9, 128.7, 123.1, 122.8, 120.9, 120.2, 116.5, 88.3, 86.3, 74.2, 70.9, 61.9. IR (KBr) λ/cm^−1^ 3352, 3106, 2926, 1630, 1575, 1424, 1219, 652. Anal. Cal. C_16_H_16_ClN_5_O_5_: C = 48.76%, H = 4.06%, Cl = 9.00%, N = 17.78%.

**Figure 7:**
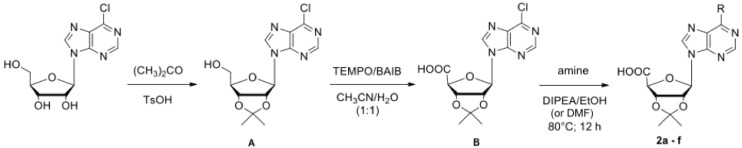
General synthesis steps of series 2.

**Figure 8:**
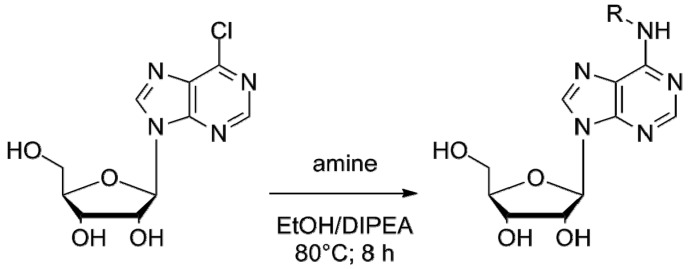
General scheme of synthesis of series 1.

Series 2: These derivatives of adenosine were obtained by nucleophilic substitution of 1′-deoxy-1′-(6-chloro-9*H*-purin-9-yl)-2′,3′-*O*-isopropylidene-beta-d-ribofuranuronic acid, obtained from successive modifications of the commercial precursor 6-chloropurine riboside: protection of vicinal diols 2′-OH and 3′-OH [42] and, then, total oxidation of 5′-OH group [43]. Figure 7 shows the general procedure and reaction conditions.

200 mg (0.7 mmol) of the commercial precursor 6-chloropurine riboside and acetone (10 mL) were mixed under agitation at room temperature for 30 min. This was followed by the slow addition of *p*-toluensulfonic acid (5.57 mmol). The solution was kept under stirring conditions at room temperature for 3 h. The progress of the reaction was monitored by TLC. Sodium bicarbonate (1.5 g) was added and maintained under agitation. Once the reaction was finished, the solid phase was removed and washed with ethyl acetate (×2). The product was then purified by column chromatography with mixtures of CH_2_Cl_2_-MeOH, obtaining the compound *[6-(6-Chloro-purin-9-yl)-2,2-dimethyl-tetrahydro-furo[3,4-d][1,3]dioxol-4-yl]-methanol* (**A**); Yellow solid, 74.1% yield; m.p. 155–158 °C ^1^H–NMR (CDCl_3_, 400.1 MHz)*δ*: 8.72 (s, 1H, CH-Ar purine); 8.31 (s, 1H, CH-Ar purine); 6.01 (d, *J* = 8.0 Hz, 1H, CH-1′); 5.16 (m, 1H, CH-2′); 4.97 (d, *J* = 7.83 Hz, 1H, CH-3′); 4.52 (d, *J* = 1.22 Hz, 1H, CH-4′); 3.83 (m, 2H, CH_2_-5′); 5.06 (m, 1 OH); 1.62 (s, 3H, ketal); 1.35 (s, 3H, ketal). ^13^C–NMR (CDCl_3_, 100.6 MHz) *δ*: 151.6, 151.4, 148.8, 144.4, 132.5, 114.0, 93.4, 86.2, 83.2, 81.1, 62.7, 27.1, 24.8. IR (KBr) λ/cm^−1^ 3320, 2906, 2863, 959, 733. Anal. Cal. C_13_H_15_ClN_4_O_4_: C = 47.75%, H = 4.59%, Cl = 10.85%, N = 17.14%.

For oxidation of 5’–OH, 100 mg of **A** (0.31 mmol) were added to H_2_O/CH_3_CN (1:1 mixture) and placed in an ultrasound bath for 30 min. The solvent was removed by vacuum, and the residue obtained was stirred with diethyl ether (50 mL), filtered and then dried before being purified by a SiO_2_ column chromatography with mixtures of CH_2_Cl_2_-MeOH, obtaining *6-(6-Chloro-purin-9-yl)-2,2-dimethyl-tetrahydro-furo[3,4-d][1,3]dioxole-4-carboxylic acid* (**B**), yellow solid, 80.5% yield; m.p. 209–211 °C. ^1^H–NMR (DMSO-*d*_6_, 400.1 MHz) *δ*: 9.20 (s, 1H, CH-Arpurine); 8.73 (s, 1H, CH-Ar purine); 6.31 (s, 1H, CH-1′); 5.28 (d, *J* = 5.62, 1H, CH-2′); 5.20 (d, *J* = 5.62 Hz, 1H, CH-3′); 4.54 (s, 1H, CH-4′); 1.52 (s, 3 H, ketal); 1.31 (s, 3H, ketal). ^13^C–NMR (DMSO-*d*_6_, 100.6 MHz) *δ*: 173.1, 151.9, 150.9, 149.1, 147.3, 131.4, 112.8, 91.7, 88.2, 84.9, 84.4, 27.2, 25.5. IR (KBr) λ/cm^−1^ 3409, 2990, 2937, 1592, 1336, 1206, 1086, 635. Anal. Cal. C_13_H_13_ClN_4_O_5_: C = 45.79%, H = 3.82%, Cl = 10.40%, N = 16.44%.

Compound **B** was fused with amines by nucleophilic aromatic substitution giving the series 2 of compounds:

*6-[6-(2-Hydroxy-phenylamino)-purin-9-yl]-2,2-dimethyl-tetrahydro-furo[3,4-d][1,3]dioxole-4-carboxylic acid* (**2a**): was prepared following the above describe procedure starting from **B** and 2-aminephenol gray solid, 43.4% yield; m.p. 275–277 °C. ^1^H–NMR (DMSO-*d*_6_, 400.1 MHz) *δ*: 8.91 (s, 1H, CH-Ar purine); 8.89 (s, 1H, OH-Ar); 8.42 (s, 1H, CH-Ar purine); 8.32 (d, *J* = 7.58 Hz, 1H, CH-Ar); 6.87 (m, 3H, CH-Ar); 6.25 (s, 1H, CH-1′); 5.15 (m, 2H, CH-2′, CH-3′); 4.47 (s, 1H, CH-4′); 1.51 (s, 3H, CH_3_-ketal); 1.30 (s, 3H,CH_3_-ketal). ^13^C–NMR (DMSO-*d*_6_, 100.6 MHz) *δ*: 173.1, 152.5, 152.0, 149.4, 142.0, 127.8, 127.8, 123.8, 121.0, 120.2, 118.9, 115.7, 112.9, 90.8, 87.8, 85.2, 84.5, 27.4, 25.6. IR (KBr) λ/cm^−1^ 3407, 2984, 2935, 1624, 1219, 1092. Anal. Cal. C_19_H_19_N_5_O_6_: C = 55.16%, H = 4.60%, N = 16.93%.

*6-[6-(2-Hydroxy-5-methyl-phenylamino)-purin-9-yl]-2,2-dimethyl-tetrahydro-furo[3,4-d][1,3]dioxole-4-carboxylic acid* (**2b**): was prepared following the above describe procedure, starting from **B** and 2-amine-4-methylphenol. Black solid amorphous, 98.5% yield; m.p. 247–252 °C. ^1^H–NMR (DMSO-*d*_6_, 400.1 MHz) *δ*:12.46 (s, 1H, 5′-COOH); 8.91 (s, 1H, OH-Ar); 8.50 (s, 1H, CH-Ar purine); 8.43 (d, *J* = 4.00 Hz, 1H, CH-Ar); 8.42 (s, 1H, CH-Ar purine); 8.23 (d, *J* = 7.83 Hz, 1H, CH-Ar); 8.20 (s, 1H, CH-Ar); 6.67 (d, *J* = 7.83 Hz, 1H, CH-Ar); 6.24 (s, 1H, CH-1′); 5.14 (d, *J* = 12.72 Hz, 2H, CH-2′, CH-3′); 4.46 (s, 1H, CH-4′); 2.22 (s, 3H, CH_3_-Ar); 1.52 (s, 3H, CH_3_-ketal); 1.30 (s, 3H, CH_3_-ketal). ^13^C–NMR (DMSO-*d*_6_, 100.6 MHz) *δ*: 173.1, 152.6, 151.9, 149.3, 146.0, 127.6, 127.3, 123.9, 121.1, 120.2, 115.4, 112.2, 90.9, 87.8, 85.2, 84.5, 65.4, 27.5, 25.6, 21.2. IR (KBr) λ/cm^−1^ 3468, 2987, 2926, 1627, 1216, 1092. Anal. Cal. C_20_H_21_N_5_O_6_: C = 56.15%, H = 4.91%, N = 16.38%.

*6-[6-(4-Amino-phenylamino)-purin-9-yl]-2,2-dimethyl-tetrahydro-furo[3,4-d][1,3]dioxole-4-carboxylic acid* (**2c**): was prepared following the above describe procedure starting from **B** and 1,4-phenylendiamine. Black solid, 95.6% yield; m.p. 250–253 °C. ^1^H–NMR (DMSO-*d*_6_, 400.1 MHz) *δ*: 9.32 (s, 1H, NH); 8.83 (s, 1H, CH-Ar); 8.23 (s, 1H, CH-Ar purine); 7.40 (d, *J* = 8.56 Hz, 2H, CH-Ar); 6.55 (d, *J* = 8.56 Hz, 2H, CH-Ar) 6.19 (s, 1H, CH-1′); 5.10 (m, 2H, CH-2′, CH-3′); 4.43 (s, 1H, CH-4′); 3.64 (s, 3H, CH_3_-ketal); 3.37 (s, 3H, CH_3_-ketal); 3.15 (s, 2H, NH_2_). ^13^C–NMR (DMSO-*d*_6_, 100.6 MHz) *δ*: 172.9, 152.6, 152.4, 149.2, 145.1, 140.8, 128.5, 123.4, 119.4, 113.9, 112.7, 90.4, 87.4, 84.9, 84.2, 27.3, 25.4. IR (KBr) λ/cm^−1^ 3447, 3213, 3109, 2987, 2932, 1624, 1514, 2095, 1213. Anal. Cal. C_19_H_20_N_6_O_5_: C = 55.30%, H = 4.85%, N = 20.37%. 

*6-[6-(5-Amino-2H-[1,2,4]triazol-3-ylsulfanyl)-purin-9-yl]-2,2-dimethyl-tetrahydro-furo[3,4-d][1,3]dioxole-4-carboxylic acid* (**2d**): was prepared following the above describe procedure starting from **B** and 3-amine-1,2,4-triazol-5-tiol. Ocher solid, 48.2% yield; m.p. 142–145 °C. ^1^H–NMR (DMSO-*d*_6_, 400.1 MHz) *δ*: 12.25 (s, 1H, -COOH); 8.78 (s, 1H, CH-Ar); 8.56 (s, 1H, CH-Ar); 6.38 (s, 1H, CH-1′); 6.16 (m, 2H, NH_2_); 5.81 (s, 1H, NH); 5.46 (d, *J* = 5.62 Hz, 1H, CH-2′); 5.37 (d, *J* = 5.38, 1H, CH-3′); 4.62 (d, *J* = 8.80, 1H, CH-4′); 1.55 (s, 3H, CH_3_-ketal); 1.33 (s, 3H, CH_3_-ketal). ^13^C–NMR (DMSO-*d*_6_, 100.6 MHz) *δ*: 172.5, 166.8, 163.0, 152.8, 151.7, 149.1, 145.2, 130.7, 112.7, 90.5, 84.8, 84.2, 83.9, 26.8, 25.2. IR (KBr) λ/cm^−1^ 3566, 3389, 2984, 2936, 1643, 1574, 1095. Anal. Cal. C_15_H_16_N_8_O_5_S: C = 42.82%, H = 3.81%, N = 26.64%, S = 7.61%.

*6-[6-(3-Amino-phenylamino)-purin-9-yl]-2,2-dimethyl-tetrahydro-furo[3,4-d][1,3]dioxole-4-carboxylic acid* (**2e**): was prepared following the above describe procedure starting from **B** and 1,3-phenylendiamine. Purple solid, 60.8% yield; m.p. 193–195 °C. ^1^H–NMR (DMSO-*d*_6_, 400.1 MHz) *δ*:9.43 (s, 1H, NH); 8.39 (m, 1H, CH-Ar purine); 8.78 (s, 1H, CH-Ar purine); 7.20 (s, 1H, CH-Ar); 6.93 (m, 1H, CH-Ar); 6.64 (t, *J* = 7.76, 1H, CH-Ar); 6.25 (m, 1H, CH-Ar); 5.83 (m, 1H, CH-1′), 5.74 (m, 1H, CH-2′); 5.21 (m, 1H, CH-3′); 4.50 (m, 1H, CH-4′); 3.15 (s, 2H, NH_2_); 1.55 (s, 3H, CH_3_-ketal); 1.29 (s, 3H, CH_3_-ketal). ^13^C–NMR (DMSO-*d*_6_, 100.6 MHz) *δ*: 152.55, 152.39, 149.55, 149.22, 140.55, 132.45, 129.08, 120.12, 112.89, 109.54, 107.15, 103.56, 100.47, 90.64, 84.97, 84.58, 45.56, 27.38, 25.57. IR (KBr) λ/cm^−1^ 3450, 3213, 3114, 2987, 2932, 1636, 1219, 1097. Anal. Cal. C_19_H_20_N_6_O_5_: C = 55.30%, H = 4.85%, N = 20.37%. 

*6-[6-(5-Chloro-2-hydroxy-phenylamino)-purin-9-yl]-2,2-dimethyl-tetrahydro-furo[3,4-d][1,3]dioxole-4-carboxylic acid* (**2f**): was prepared following the above describe procedure starting from **B** and 2-amine-4-chlorophenol. Brown solid, 75.3% yield; m.p. 247–251 °C. ^1^H–NMR (DMSO-*d*_6_, 400.1 MHz) *δ*: 8.90 (s, 1H, OH-Ar); 8.56 (s, 1H, CH-Ar purine); 8.48 (s, 1H, CH-Ar purine); 6.95 (d, *J* = 8.31 Hz, 2H, CH-Ar); 6.80 (d, *J* = 8.31 Hz, 1H, CH-Ar); 6.25 (s, 1H, CH-1′); 5.18 (m, 1H, CH-2′); 5.09 (d, *J* = 5.14 Hz, 1H, CH-3′); 4.44 (s, 1H, CH-4′); 1.51 (s, 3H, CH_3_-ketal); 1.30 (s, 3H, CH_3_-ketal). ^13^C–NMR (DMSO-*d*_6_, 100.6 MHz) *δ*: 172.9, 152.5, 151.5, 149.4, 142.4, 129.3, 122.4, 120.4, 118.9, 116.2, 115.5, 114.9, 112.8, 90.88, 87.9, 85.1, 84.5, 27.4, 25.6. IR (KBr) λ/cm^−1^ 3434, 2925, 2849, 1623, 1209, 1085, 646. Anal. Cal. C_19_H_18_ClN_5_O_6_: C = 52.32%, H = 4.13%, Cl = 8.13%, N = 16.06%.

### 3.5. Biological Activity

#### 3.5.1. Cancer Cell Line and Cell Culture Conditions

Gastric cancer cell lines (AGS) were obtained from the American Type Culture Collection (ATCC). Cells were cultured in the recommended media (RPMI-1640). The culture medium was supplemented with 10% FBS, 5 µg/mL of Plasmocin (prophylactic), 250 µg/mL of amphotericin-B and 25 µg/mL of gentamicin. The cell line was maintained at a 37 °C, 5% CO_2_ and with a humidity of 95%.

#### 3.5.2. General Screening of Adenosine Derivatives

Gastric cancer AGS cells were exposed to different concentrations of semisynthetic compounds (series 1 and series 2; 10 nM, 100 nM and 1000 nM) for 72 h. Each experiment was repeated twice. This procedure allowed us to select those compounds with higher activity, which was expressed as a percentage of inhibition of proliferation compared to the vehicle.

A commercial agonist (IB-MECA), a noselective adenosine agonist (Ado), the commercial precursor for semisynthesis (6-chloropurine riboside), as well as a selective antagonist (MRS-1523) were included as controls.

#### 3.5.3. Theoretical Evaluation of ADME Properties

Preliminary data for an ADME profile analysis was estimated using the Molinspiration property calculation program. Molecular properties such as partition coefficient (Log P), topological polar surface area (TPSA), hydrogen bond donors and acceptors, rotatable bonds, number of atoms, molecular weight, and violations of Lipinski’s rule of five, were calculated for each compound [35].

#### 3.5.4. Statistical Analysis

For determining biological activity, each experiment was performed in duplicate. Statistical analysis of data was carried out using the Student *t*-test. *p* < 0.05 was considered statistically significant.

## 4. Conclusions

We describe the synthesis and structural determination of new adenosine derivatives. These compounds were obtained through nucleophilic aromatic substitution reactions (S_N_Ar) by coupling commercially available chloropurine riboside to different amines. Overall, the yields were moderate to good. Biological activity assays revealed several potential compounds with antiproliferative effects that do not violate Lipinski’s rules. New modifications at the *N*^6^-position of adenosine can therefore be an important alternative in the search of more selective and potent compounds with antiproliferative potential.

## Figures and Tables

**Table 1:** Binding energies and free energies (∆G_b_) for the fourteen compounds that were post-filtered according to their interactions with the active site of the A_3_ receptor.

Ligands	Energies	
	Binding Energy (Kcal/mol)	∆G_b_ (Kcal/mol)
**1**	−44.94	−84.71
**2**	−41.69	−79.75
**3**	−38.72	−78.28
**4**	−40.73	−77.74
**5**	−44.56	−76.52
**6**	−43.68	−76.07
**7**	−38.71	−70.76
**8**	−37.88	−69.60
**9**	−30.06	−68.64
**10**	−38.95	−68.29
**11**	−46.53	−67.00
**12**	−35.32	−66.36
**13**	−37.20	−64.83
**14**	−37.41	−57.93
**IB-MECA**	−47.29	−67.391
**MRS-1523**	−23.46	−77.363

**Table 2:** Theoretical ADME properties of the most active adenosine derivatives.

Compound	Log P	Molecular Weight	TPSA	n-ON Acceptors	n-OHNH Donors	Volume
1e	1.09	358.36	151.58	10	6	302.34
1h	1.70	403.39	144.02	11	4	342.14
2b	2.11	426.43	140.87	11	3	358.53
2f	2.85	447.83	140.87	11	3	353.32

Log P octanol/water partition coefficient; TPSA topological polar surface area; n-ON number of hydrogen acceptors; n-OHNH number of hydrogen bond donors. The data was determined with molinspiration calculation software [34].
